# Acidic mammalian chitinase is a proteases-resistant glycosidase in mouse digestive system

**DOI:** 10.1038/srep37756

**Published:** 2016-11-24

**Authors:** Misa Ohno, Masahiro Kimura, Haruko Miyazaki, Kazuaki Okawa, Riho Onuki, Chiyuki Nemoto, Eri Tabata, Satoshi Wakita, Akinori Kashimura, Masayoshi Sakaguchi, Yasusato Sugahara, Nobuyuki Nukina, Peter O. Bauer, Fumitaka Oyama

**Affiliations:** 1 Department of Chemistry and Life Science, Kogakuin University, Hachioji, Tokyo 192-0015, Japan; 2 Laboratory for Structural Neuropathology, RIKEN Brain Science Institute, Wako, Saitama 351-0198, Japan; 3 Department of Neuroscience for Neurodegenerative Disorders, Juntendo University Graduate School of Medicine, Tokyo 113-8421, Japan; 4 Laboratory of Structural Neuropathology, Doshisha University Graduate School of Brain Science, Kyotanabe-shi, Kyoto 610-0394 Japan; 5 Department of Neuroscience, Mayo Clinic, Jacksonville, FL 32224, USA

## Abstract

Chitinases are enzymes that hydrolyze chitin, a polymer of β-1, 4-linked *N*-acetyl-D-glucosamine (GlcNAc). Chitin has long been considered as a source of dietary fiber that is not digested in the mammalian digestive system. Here, we provide evidence that acidic mammalian chitinase (AMCase) can function as a major digestive enzyme that constitutively degrades chitin substrates and produces (GlcNAc)_2_ fragments in the mouse gastrointestinal environment. AMCase was resistant to endogenous pepsin C digestion and remained active in the mouse stomach extract at pH 2.0. The AMCase mRNA levels were much higher than those of four major gastric proteins and two housekeeping genes and comparable to the level of pepsinogen C in the mouse stomach tissues. Furthermore, AMCase was expressed in the gastric pepsinogen-synthesizing chief cells. The enzyme was also stable and active in the presence of trypsin and chymotrypsin at pH 7.6, where pepsin C was completely degraded. Mouse AMCase degraded polymeric colloidal and crystalline chitin substrates in the gastrointestinal environments in presence of the proteolytic enzymes. Thus, AMCase can function as a protease-resistant major glycosidase under the conditions of stomach and intestine and degrade chitin substrates to produce (GlcNAc)_2_, a source of carbon, nitrogen and energy.

Chitin, a linear polymer of β-1, 4-linked *N*-acetyl-D-glucosamine (GlcNAc), is the second most abundant natural polysaccharide in nature and functions as a major structural component in fungi, crustaceans and insects^1^. Chitin has an important role as a primary source of carbon, nitrogen and energy for organisms producing chitinases, such as bacteria^2^. Since chitin has been thought to be not degraded in the mammalian digestive system, it is sometimes included in animal feeds as dietary fiber^3^.

Chitinases hydrolyze the β-1, 4 glycoside bonds of chitin. Although mice and humans do not synthesize chitin and its synthase, they do express two active chitinases, chitotriosidase (Chit1) and acidic mammalian chitinase (AMCase)^1^,^4^. Chit1 levels are markedly increased in the plasma of patients with Gaucher disease, an autosomal recessive lysosomal storage disorder^5^. This enzyme is the first purified and cloned mammalian chitinase^6^,^7^. AMCase is the second chitinase and acquired the name for its acidic isoelectric point^8^.

AMCase has attracted substantial attention due to its expression fluctuations under certain pathological conditions such as asthma, allergic inflammation, ocular allergy, dry eye syndrome and gastric cancer^9^,^10^,^11^,^12^,^13^,^14^,^15^,^16^. AMCase is an important downstream effector of interleukin-13 stimulation in T Helper-2 (Th2) cell-mediated immune responses to ovalbumin, pathogens and parasites^9^,^17^. AMCase also functions as a critical initiator of protective type 2 responses to intestinal nematodes^18^. In addition, several genetic variants of AMCase are associated with bronchial asthma in humans^19^,^20^,^21^,^22^.

Murine AMCase is most active at pH of around 2^8^,^23^,^24^,^25^. Mouse stomach produces large quantities of AMCase mRNA and protein^26^,^27^. Thus, AMCase seems to function as a digestive enzyme that breaks down chitin-containing ingested material in mouse stomach. However, it has not been known whether it has this role throughout the gastrointestinal tract (GIT). Here, we show that AMCase is a major chitinase in stomach resistant to pepsin and trypsin/chymotrypsin digestion and that it can function as a digestive enzyme under conditions also in other parts of GIT.

## Results

### AMCase is resistant to endogenous pepsin C digestion

Pepsin C is autocatalytically converted from pepsinogen C, the secreted inactive zymogen, at acidic conditions of the gastric fluid^28^,^29^. First, we examined whether murine endogenous AMCase is resistant to digestion by endogenous pepsin C in artificially created mouse stomach environment at pH 2.0 and 37 °C. Soluble protein fraction was prepared from mouse gastric tissue in the absence of protease inhibitor and incubated at pH 7.6 or pH 2.0 for up to 60 min.

**Figure 1:**
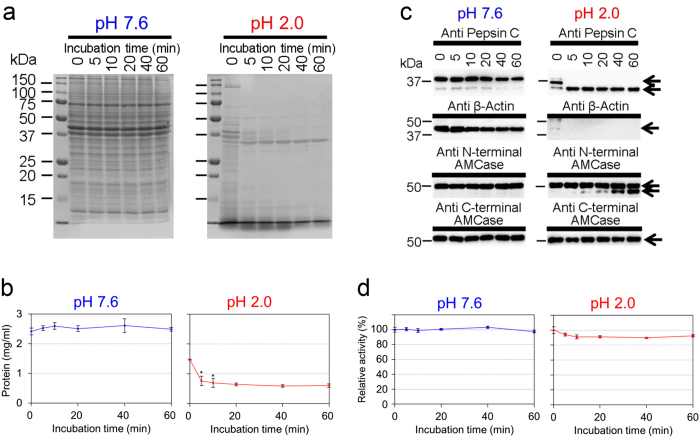
AMCase is not degraded by pepsin. Soluble proteins obtained from mouse stomach were incubated at 37 °C for 0, 5, 10, 20, 40 and 60 min at pH 7.6 or 2.0. (**a**) Total protein analysis by Coomassie Brilliant Blue (CBB) staining, (**b**) Total protein levels quantification, (**c**) Western blot and (**d**) chitinolytic activity assay measured at pH 2.0 as described in Methods. Values in (**b**) and (**d**) represent mean ± SD from a single experiment conducted in triplicate. *p < 0.05. P-values were determined using Student’s t-test.

At pH 7.6, no changes in the band pattern and intensities were noticeable during the 60 min incubation (Fig. 1a and b, left panels). In contrast to the pH 7.6 condition, we observed a time-dependent decrease of the soluble proteins with a marked reduction after as early as 5 min of incubation at pH 2.0, although several bands remained unmodified within 60 min of incubation (Fig. 1a and b, right panels). Western blot analysis indicated that the conversion from pepsinogen C to pepsin C occurred within first 5 min of incubation at pH 2.0 (Fig. 1c, upper right panel and Supplementary Fig. S1). Beta-actin, a soluble protein, was stably present during the 60 min of incubation at pH 7.6 (Fig. 1c and Supplementary Fig. S1), whereas it was only slightly detectable at pH 2.0 at time 0 and degraded completely within first 5 min of incubation (Fig. 1c).

Mouse AMCase is composed of the N-terminal catalytic domain (CatD) and the C-terminal chitin-binding domain (CBD)^8^ (Supplementary Figs S2 and S3). It has been suggested that CBD recognizes chitin and CatD degrades it^30^. Anti N-terminal AMCase antibody (Supplementary Fig. S3) recognized two bands at 50 and 41 kDa. The 50 kDa band was not significantly affected during the 60 min incubation at pH 7.6, whereas it was slightly decrease during the incubation at pH 2.0 (Fig. 1c and Supplementary Fig. S1). The 41 kDa band was present at pH 2.0 and its intensity gradually increased after 10 min onward (Fig. 1 and Supplementary Fig. S1). Anti C-terminal AMCase antibody (Supplementary Fig. S3) recognized the 50 kDa band, which was slightly decreased during the incubation at any of the pH conditions (Fig. 1c and Supplementary Fig. S1). The anti C-terminal AMCase antibody did not detect the CBD fragment (Supplementary Fig. S4). The results clearly indicate that the pepsin-resistant 41 kDa band represents CatD^25^ and the resulting CBD was degraded by pepsin suggesting that, in contrast to other soluble proteins, AMCase is stable in the presence of pepsin C at pH 2.0, even though it is partly cleaved, releasing the CatD fragment. Moreover, the chitinolytic activity at pH 2.0 (optimal pH of AMCase) remained virtually unchanged during the 60 min incubation at either of the pH conditions (Fig. 1d), indicating that the chitinolytic activity is pepsin-resistant.

### AMCase is a major gastric protein and produced in pepsinogen C-synthesizing chief cells

**Figure 2:**
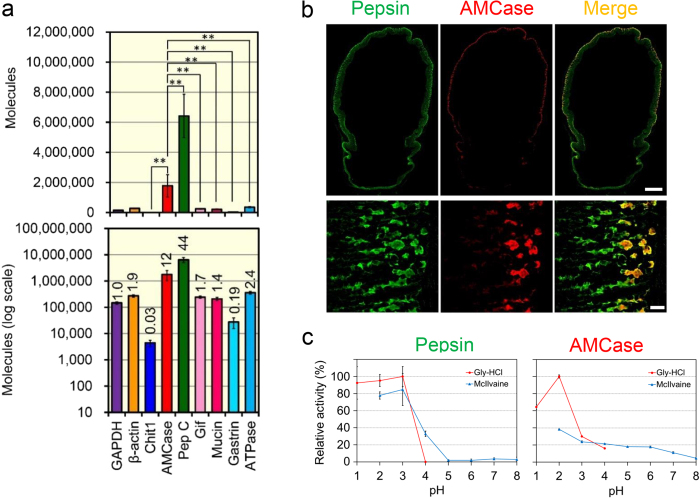
Expression, localization and activity of pepsin C and AMCase in mouse stomach. (**a**) The mRNA levels of nine genes were quantified by qPCR. Upper panel, actual value; lower panel, logarithmic scale. Y axis represents molecules per 10 ng of total RNA. The numbers in the figure indicate relative expression levels with GAPDH set at 1.0. Pep C, pepsinogen C; Gif, gastric intrinsic factor; ATPase, H^+^/K^+^-ATPase. (**b**) Expression and co-localization of pepsin C and AMCase proteins in mouse stomach sections shown by immunohistochemistry using anti-pepsin C (green) and anti-human AMCase (red). Scale bars, 1,000 μm (upper panels); 50 μm (lower panels). (**c**) pH profile of pepsin and AMCase. Values in (**a**) and (**c**) represent mean ± SD. **p < 0.01. P-values were determined using Student’s t-test.

In stomach, number of gastric mucosa proteins, such as gastric intrinsic factor^31^, mucin^32^, gastrin^33^ and H^+^/K^+^-ATPase^34^, are synthesized by various glandular cells. We compared the expression levels of AMCase mRNA with those of the gastric mucosa proteins and housekeeping genes, glyceraldehyde-3-phosphate dehydrogenase (GAPDH) and β-actin, using our previously reported quantitative PCR system^26^,^27^. We found that AMCase was the second most abundantly expressed molecule in the stomach tissue being exceeded only by pepsinogen C and its level was significantly higher those of Chit1 and other tested gastric mucosa and housekeeping genes mRNAs (Fig. 2a). These results indicate that AMCase is a major transcript in the mouse stomach.

Pepsinogen C is synthesized in gastric chief cells^28^,^29^. Using double immunostaining, we found that AMCase is also expressed in these cells, although not all pepsin C-positive cells are immunoreactive to AMCase (Fig. 2b).

The mature protein, pepsin C, then exhibits maximum activity at pH 2.0^28^,^29^. We observed maximum activity of pepsin C at pH 2~3 which is lost at pH > 5.0 (Fig. 2c, left). AMCase also exhibited maximum activity at pH 2.0, but in contrast to pepsin C, its activity was to certain level preserved at more neutral conditions (pH up to 7.0) (Fig. 2c, right). Thus, AMCase is a major gastric protein with chitinolytic activity and resistance to pepsin C in the mouse stomach environment.

### AMCase is resistant to digestion by trypsin and chymotrypsin

**Figure 3:**
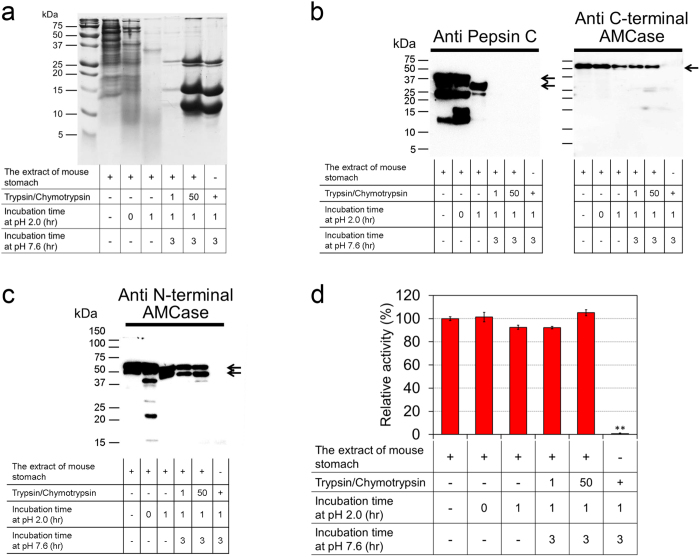
AMCase is resistant to digestion by trypsin and chymotrypsin. Soluble proteins fraction from mouse stomach was incubated under stomach-like condition, followed by incubation in intestine-like environment containing trypsin and chymotrypsin at equal or 50-fold excess to the amount of the soluble proteins. The samples were analyzed by (**a**) CBB staining, (**b**,**c**) Western blot and (**d**) measurement of chitinolytic activity. All incubations were performed at 37 °C. Values represent mean ± SD. **p < 0.01 P-values were determined using Student’s t-test.

Stomach empties its content that includes pepsin C and AMCase into the intestinal lumen where the acid is neutralized by sodium bicarbonate and where trypsin and chymotrypsin are secreted. Here, the predigested proteins are further degraded into smaller peptides. We artificially generated intestinal environment and investigated the stability of AMCase in the presence of trypsin and chymotrypsin. We incubated the soluble protein fraction from mouse stomach at pH 2.0 and 37 °C for 1 hour, followed by incubation under intestine-like neutral condition (pH 7.6) in the presence of equal or 50-fold higher levels of trypsin and chymotrypsin at 37 °C for 3 hours. In agreement to the previous experiment (Fig. 1), the total protein quantity drastically decreased during the incubation at pH 2.0. Subsequent incubation at pH 7.6 in the presence of trypsin and chymotrypsin resulted in further degradation of total protein (Fig. 3a). Pepsin C was completely digested, whereas AMCase levels were not affected in the presence of equal amounts of trypsin and chymotrypsin (Fig. 3b and Supplementary Fig. S5). Anti C-terminal AMCase antibody recognized the 50 kDa band, which was decreased during the incubation at stomach pH conditions. However, AMCase level was not reduced by the equal or 50-fold excess of both enzymes (Fig. 3b and Supplementary Fig. S5). Using the anti-N-terminal AMCase antibody, we also detected the 41 kDa band in addition to that of 50 kDa, which were not altered significantly by the trypsin and chymotrypsin (Fig. 3c and Supplementary Fig. S5). Moreover, the chitinolytic activity was not significantly affected by any of the incubation conditions (Fig. 3d). These data indicate that AMCase and its cleaved CatD are stable even in the presence of trypsin and chymotrypsin at pH 7.6.

### AMCase degrades chitin substrates in the gastrointestinal environments in presence of the proteolytic enzymes

**Figure 4:**
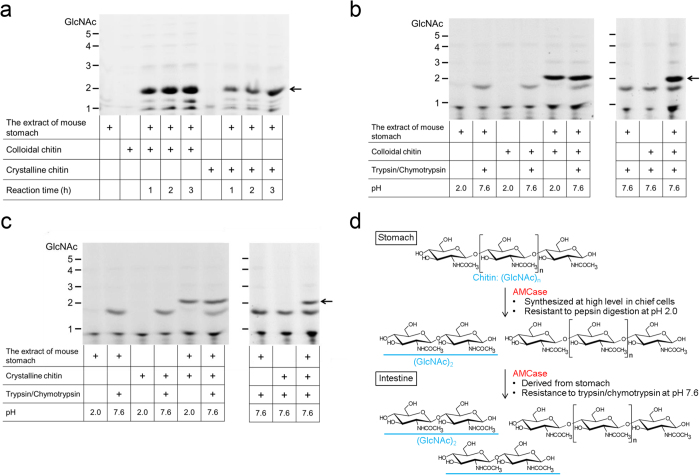
Chitin is degraded in stomach and intestine environment. (**a**) Colloidal and crystalline chitin substrates were degraded by the stomach extract containing AMCase during 1–3 hours incubation. (**b**) and (**c**) Degradation products were generated during incubation of colloidal (**b**, left) and crystalline chitin (**c**, left) with the extract of mouse stomach at pH 2.0 for 1 hour and subsequently at pH 7.6 for 3 hours after addition of equal amount of trypsin and chymotrypsin. Additionally, colloidal (**b**, right) and crystalline chitin (**c**, right) were degraded when substrates were reacted at pH 7.6 for 3 hours after addition of equal amount of trypsin and chymotrypsin. All incubations were performed at 37 °C. (**d**) Summary of the results in this study. Mouse AMCase degrades chitin substrates to (GlcNAc)_2_ in both gastric and intestinal environments.

Finally, we tested whether AMCase can degrade chitin polymer in both gastric and intestinal environments. We incubated chitin substrates with the mouse stomach extract and the resulting mono- and oligosaccharides were covalently labeled at their reducing end-groups with a fluorophore and separated by high-resolution polyacrylamide gel electrophoresis (PAGE)^8^,^24^,^25^,^35^ as described in Methods. The endogenous AMCase degraded both colloidal and crystalline chitin at pH 2.0 as early as after 1 hour incubation producing primarily (GlcNAc)_2_ fragments (Fig. 4a). The substrates were similarly digested in the presence of trypsin and chymotrypsin during both subsequent (pre-incubation at pH 2.0) and a single incubation step at pH 7.6 (Fig. 4b and c). The levels of (GlcNAc)_2_ from both chitin substrates were a little higher after incubation in intestinal environment as compared to the stomach conditions (Fig. 4b and c). These results confirm that mouse AMCase degrades polymeric colloidal as well as crystalline chitin to (GlcNAc)_2_ in both gastric and intestinal environments (Fig. 4d).

## Discussion

Here, we show that AMCase is resistant to proteolytic activity of pepsin C at pH 2.0 as well as trypsin and chymotrypsin at pH 7.6 and retains its chitinolytic ability under both conditions. AMCase mRNA expression is substantially higher (followed by pepsinogen C) than those of Chit1, four major gastric proteins and two housekeeping genes. Our results indicate that AMCase functions as a digestive enzyme in the whole mouse GIT.

Stomach soluble proteins including β-actin were degraded by endogenous pepsin C at pH 2.0 and 37 °C, while AMCase was resistant. Furthermore, it should be noted that while pepsin C was degraded by trypsin and chymotrypsin at the intestinal environment, full-length AMCase and its CatD were largely not affected by excessive concentrations of these enzymes. Importantly, the chitinolytic activity of AMCase was still maintained under these conditions. Our previous study has shown that the primary structure of the CatD is sufficient to form a tertiary structure required for chitinolytic activity, substrate recognition and degradation in the absence of CBD^25^. Thus, full-length AMCase as well as its CatD are able to efficiently function in different GIT parts.

Previous studies showed that mouse AMCase is most active at pH of around 2^8^,^23^,^24^,^25^ and is abundantly expressed in the stomach^8^,^23^,^26^,^27^. Using double immunostaining, we show that AMCase is produced in pepsinogen C-synthesizing chief cells in the mouse stomach tissue, which is consistent with previous studies using *in situ* hybridization^23^,^36^ or single antibody staining^37^. Wild mice eat chitin containing foods such as insects, whereas laboratory mice are fed artificial diets containing dried brewers’ yeast, which also contains chitin in the cell wall. AMCase has also been found in insectivorous bats, which eat arthropods containing chitin^38^. Bat AMCase is synthesized in the stomach chief cells and it is most active at bat stomach environment (highest between pH 5.0 and pH 6.0)^38^. Therefore, both mouse and bat AMCase can function as digestive enzymes in their stomach tissues.

Here, we show a major physiological role of AMCase acting as a digestive enzyme under GIT conditions. Recently, AMCase has been shown to have a critical role in initiating type 2 immunity against the chitin-containing nematode^18^. Thus, AMCase can function as not only a digestive enzyme but also a part of the host defense against chitin-containing pathogens in the mouse GIT.

Chitin is the second most abundant naturally occurring polysaccharide. Since it has been thought to be not degraded in the digestive system, it has been considered dietary fiber and has been included in animal feeds^3^. Our results clearly show that AMCase is able to digest chitin polymers even in the presence of pepsin C, trypsin and chymotrypsin. The main degradation product, (GlcNAc)_2_, can be then uptaken in mouse GIT, providing the primary source of carbon, nitrogen and energy. Thus, chitin can be used in feeds for murine breeding^39^.

## Methods

### Mouse stomach proteins extract preparation

C57BL/6 J mice (CLEA Japan) were bred at the RIKEN Brain Science Institute Animal Facility. All animal experiments were performed in compliance with the institutional guidelines. The protocol was approved by the Committee on the Ethics of Animal Experiments of the RIKEN Brain Science Institute (Approval No. H19-2B013). All surgeries were performed under total anesthesia by diethyl ether, and all efforts were made to minimize suffering. Stomach tissue isolated from 3-month old C57Bl/6 J mice was homogenized in 10 volumes of ice-cold TS buffer [20 mM Tris-HCl (pH7.6), 150 mM NaCl] using a Teflon/glass homogenizer. The homogenates were centrifuged at 17,000 g for 10 min at 4 °C, and the supernatants were kept. Tris-HCl buffer (pH 7.6) or Gly-HCl buffer (pH 2.0) was added at final concentration of 0.1 M. After the pre-incubation at 37 °C for 0, 5, 10, 20, 40 or 60 min, protein inhibitor (Complete Mini, Roche) was added. After incubation under the conditions of pH 2.0 at 37 °C, the solutions were neutralized by addition of 1 M Tris-HCl (pH 7.6). Then, equal or 50-fold excess amount (6 μg and 304 μg) of a 1:1 mixture of the trypsin (Sigma-Aldrich) and chymotrypsin (Sigma-Aldrich) was added to the reaction mixture. The reaction mixtures were incubated at 37 °C for 3 hours at pH 7.6.

### Antibody Preparation

Rabbit polyclonal antibodies specific to mouse N-terminal AMCase was generated by Eurofins Genomics. Cys-peptides were conjugated through the added C-terminal or N-terminal cysteine to keyhole limpet hemocyanin (KLH). Sera from immunized rabbits were affinity-purified using the antigen with Cys (mouse N-terminal AMCase, CAFNDLKNRNSKL) coupled to Sulfolink (Pierce). The specificity of each antibody was confirmed by Western blot.

### SDS-polyacrylamide gel electrophoresis, CBB staining and Western blot

The obtained protein fractions were analyzed using standard SDS-polyacrylamide gel electrophoresis (PAGE), followed by Coomassie Brilliant Blue R-250 (Sigma-Aldrich) staining or Western blot. We used All Blue (Bio-Rad Laboratories) and Dual Xtra (Bio-Rad Laboratories) as molecular weight markers. Separated proteins were transferred to a polyvinylidene fluoride (PVDF) membrane (Immobilon-P, Millipore), which was probed with polyclonal anti-human C-terminal AMCase^27^, anti-mouse N-terminal AMCase, anti- mouse C-terminal AMCase^27^, polyclonal goat anti-pepsin C (I-19) antibody (Santa Cruz) or monoclonal anti-β-actin (clone AC-15) (Sigma-Aldrich), followed by peroxidase-conjugated AffiniPure F (ab’)_2_ Fragment Donkey Anti-Rabbit IgG (H+L) (Jackson ImmunoResearch Laboratories), AffiniPure Donkey Anti-Goat IgG-HRP (Jackson ImmunoResearch laboratories) or AffiniPure Donkey Anti-Mouse IgG (H+L) (Jackson ImmunoResearch laboratories). Bound antibodies were detected using Immobilon Western Chemiluminescent HRP Substrate (Millipore). The immunoblots were analyzed and quantified using the Luminescent Image Analyzer (ImageQuant LAS 4000, GE Healthcare) according to the manufacturer’s instructions.

### Determination of protein concentration

Protein concentrations were determined by the Bradford Protein Assay (Bio-Rad) using the BioPhotometer Plus UV/Vis photometer (Eppendorf), with bovine serum albumin as a standard.

### Chitinase enzymatic assays

Chitinolytic activity was determined using the synthetic chromogenic substrate, 4-nitrophenyl *N, N*’-diacetyl-β-D-chitobioside (Sigma-Aldrich), at a concentration of 200 μM. Each reaction was performed in triplicate. All enzymatic reactions were conducted in a volume of 50 μL containing total protein extract of mouse stomach in Gly-HCl buffer (pH 1.0 to pH 4.0) or McIlvaine’s buffer (0.1 M citric acid and 0.2 M Na_2_HPO_4_; pH 2.0 to pH 8.0). After incubation at 37 °C for 20 min, 20 μL of 1 M sodium carbonate solution was immediately added to the reaction mixture. The absorbance of the released 4-nitrophenolate ion was measured at 405 nm.

### RNA and cDNA preparation

Total RNA was prepared from mouse stomach tissue using TRIzol Reagent (Invitrogen) according to the manufacturer’s instructions. To remove the trace amounts of contaminating genomic DNA, the total RNA samples were treated with RQ1 RNase-Free DNase (Promega) according to the manufacturer’s recommended protocol. The concentrations of the nucleic acids were determined by measuring the absorbance at 260 nm. Each of the total RNA samples (3 μg) was subjected to reverse transcription with random hexamers as primers. The reaction mixture (15 μl) contained the enzyme buffer [50 mM Tris-HCl (pH 8.3), 75 mM KCl, and 3 mM MgCl_2_], 100 ng of random hexamers, 10 mM dithiothreitol and 0.5 mM deoxynucleotide triphosphates (dNTPs). After heating the solution to 60 °C for 5 min and incubating the mixture at 37 °C for 5 min, 200 U of recombinant murine leukemia virus reverse transcriptase (Invitrogen) was added, and the mixture was incubated at 37 °C for 45 min. The reverse transcription was terminated by heating to 95 °C for 5 min.

### Construction of the standard DNA

Construction of the 9 genes standard DNA containing the genes of major proteins in stomach was described previously^27^. Coding sequences of gastric intrinsic factor, mucin, gastrin and H^+^/K^+^-ATPase, and template DNA were synthesized and inserted into pTAKN-2 vector by Eurofins Genomics. After ligation, these genes were amplified using the forward primer 5′-TAATACGACTCACTATAGGG-3′ and the revers primer 5′-CAGGAAACAGCTATGAC-3′. The four genes of gastric mucosa and five genes of the template DNA^26^ were digested with EcoRI ligated using T4 DNA ligase. The ligated fragments were amplified using the forward primer 5′-GTGGATTCTGTGCCGACAAAGCAGATGGCC-3′ and the reverse primer 5′-ATCATGGATACAAGTCCCGCAAAGCAGAGGCCACT-3′ and cloned into the pGEM-T Easy vector (Promega). The standard DNA (1,349 bases; see Supplementary Fig. S6) was prepared by PCR reamplification from the plasmid DNA using the same primers and was thereafter used as the standard DNA.

### Real-time PCR

Primers of gastric intrinsic factor, mucin, gastrin and H^+^/K^+^-ATPase for real-time PCR were designed based on PrimerQuest Input (Integrated DNA Technologies) and were synthesized commercially (Eurofins Genomics) (Supplementary Table. 1). PCR reactions were performed in a final volume of 13 μl containing 2 x SYBR Green Master Mix (Brilliant II SYBR Green QPCR Master Mix, Agilent), 2.7 ng of mouse cDNA or appropriate dilutions of the external standards (see below) and 2.3 pmol of the primers listed in Supplementary Table 1. Standard real-time PCR conditions for the Mx3005P system (Agilent) were used: an initial denaturation and polymerase activation step for 10 min at 95 °C, followed by 40 cycles of denaturation at 95 °C for 30 sec, annealing at 55 °C for 30 sec and polymerization at 72 °C for 10 sec. Melting curves were generated after amplification. Chit1, AMCase, pepsinogen C, GAPDH and β-actin primers have been previously reported^26^.

### Immunohistochemistry

14-week-old mice were perfused with 4% paraformaldehyde (PFA) in PBS. Stomachs were postfixed by 4% PFA in PBS overnight at 4 °C and then transferred in 30% sucrose in PBS. Stomachs were embedded in Tissue-Tek O.C.T. compound, frozen by dry ice-cooled ethanol and then cut using cryostat. 15-μm-thick sections were autoclaved for antigen retrieval and immunostained as described previously^40^. We used following primary antibodies for immunostaining: rabbit polyclonal anti-human C-terminal AMCase at 1:1,000 dilution and goat polyclonal anti-Pepsin C at 1:500 dilutions. The following secondary antibodies were used at 1:300 dilutions: Alexa Fluor 546 anti-rabbit IgG and Alexa Fluor 488 anti-goat IgG. Images were taken by BIOREVO BZ-9000 (KEYENCE) and TCS SP5 confocal microscope (Leica).

### Pepsin enzymatic assays

Pepsin activity was measured using hemoglobin from bovine blood (Sigma-Aldrich) as the substrate. In a total reaction volume of 150 μl, 1.3% (w/v) of bovine hemoglobin was reacted at 37 °C with soluble protein. After 5 min, the reaction was quenched using trichloroacetic acid (TCA) (Sigma-Aldrich) added to a final concentration of 3.2% (w/v). Samples were centrifuged and the absorbance was measured at 280 nm. We analyzed the protein fractions treated at 37 °C for 10 min.

### Degradation of colloidal and crystalline chitin by soluble protein

To inactivate Chit1 and activate pepsin, soluble protein was pre-incubated at 37 °C for 10 min. Subsequently, soluble protein was reacted with colloidal chitin and crystalline chitin. Colloidal chitin was prepared from shrimp shell chitin (Sigma-Aldrich), as described previously, and used as a substrate to determine the chitinase activity^8^,^24^. The enzymatic reactions of stomach condition using colloidal chitin (at a final concentration of 1 mg/mL) and crystalline chitin (1 mg/reaction) were performed in a volume of 50 μL containing soluble protein at pH 2.0 and 37 °C for 1 hour. In case of stomach, to inactivate Chit1 and activate pepsin, soluble protein was pre-incubated at 37 °C for 10 min. Chitin fragments generated in the stomach condition were labeled covalently at their reducing end groups with the fluorophore 8-aminonaphthalene-1, 3, 6-trisulphonic acid (ANTS, Sigma-Aldrich), and the resulting fluorescent derivatives were separated by high-resolution PAGE, as described by Jackson^35^. In case of intestine-like neutral condition, following the enzymatic reactions of stomach condition in a volume of 20 μL, we added 6 μL of 1 M Tris-HCl (pH7.6) and 6 μg of a 1:1 mixture of trypsin/chymotrypsin and reacted at pH 7.6 and 37 °C for 3 hours. Generated chitin fragments were labeled essentially as described above except for the addition of 5 μL of glacial acetic acid before ANTS labeling. *N*-acetyl chitooligoaccharides (Seikagaku Corporation) were used as a standard.

### Statistical analysis

Biochemical data were compared by Student’s t-test.

## Additional Information

**How to cite this article**: Ohno, M. *et al*. Acidic mammalian chitinase is a proteases-resistant glycosidase in mouse digestive system. *Sci. Rep.* **6**, 37756; doi: 10.1038/srep37756 (2016).

**Publisher’s note:** Springer Nature remains neutral with regard to jurisdictional claims in published maps and institutional affiliations.

## Supplementary Material

Supplementary Information
