# Supplementary material for: Acidic mammalian chitinase is a proteases-resistant glycosidase in mouse digestive system
Source: Sci Rep. 2016 Nov 24;6:37756. doi: 10.1038/srep37756 (PMC5121897; doi:10.1038/srep37756)
Supplement: Supplementary Information [file srep37756-s1.pdf]

Supplementary Information

Acidic mammalian chitinase is a proteases-resistant glycosidase in mouse digestive system

Misa Ohno[1,†], Masahiro Kimura[1,†], Haruko Miyazaki[2,3,4,†], Kazuaki Okawa[1], Riho Onuki[1], Chiyuki Nemoto[1], Eri Tabata[1], Satoshi Wakita[1], Akinori Kashimura[1], Masayoshi Sakaguchi[1], Yasusato Sugahara[1], Nobuyuki Nukina[2,3,4], Peter O. Bauer[5] and Fumitaka Oyama[1,*]

[1]Department of Chemistry and Life Science, Kogakuin University, Hachioji, Tokyo 192-0015, Japan, [2]Laboratory for Structural Neuropathology, RIKEN Brain Science Institute, Wako, Saitama 351-0198, Japan, [3]Department of Neuroscience for Neurodegenerative Disorders, Juntendo University Graduate School of Medicine, Tokyo 113-8421, Japan, [4]Laboratory of Structural Neuropathology, Doshisha University Graduate School of Brain Science, Kyotanabe-shi, Kyoto 610-0394 Japan and [5]Department of Neuroscience, Mayo Clinic, Jacksonville, FL 32224, USA

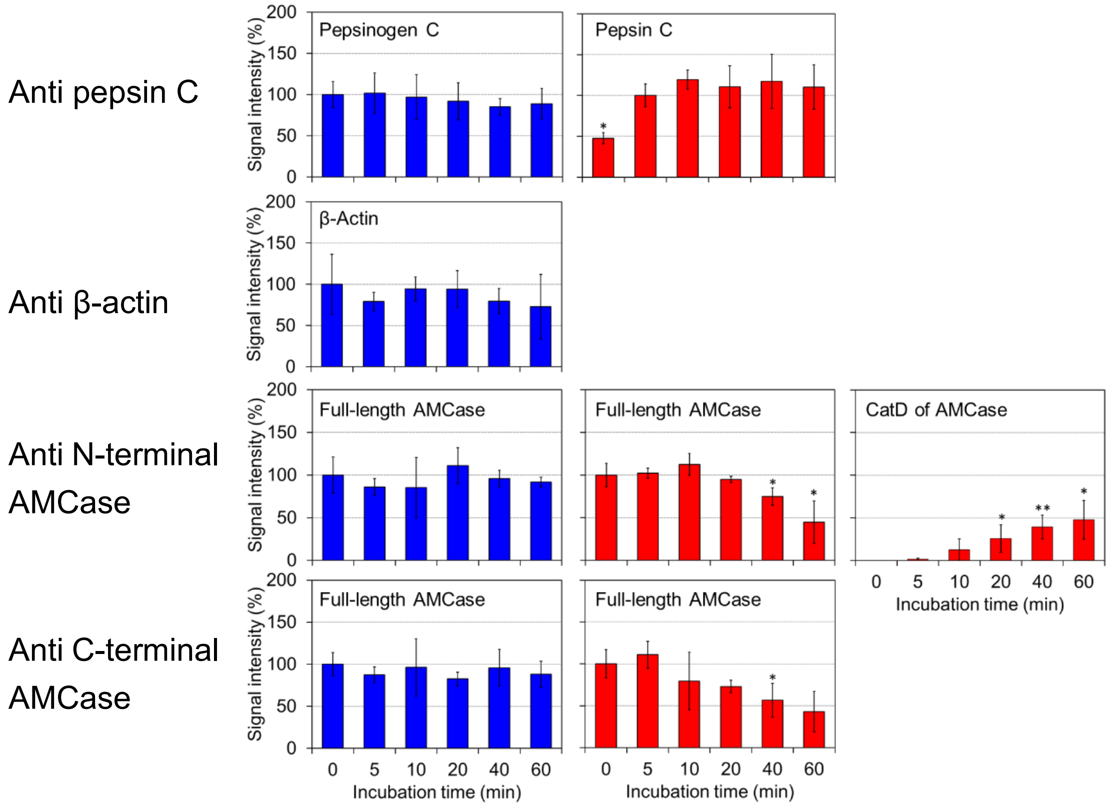

Supplementary Fig. S1. Changes of protein levels in the Western blot in Fig. 1c. Soluble proteins obtained from mouse stomach were incubated at pH 7.6 (blue bars) or 2.0 (red bars) and carried out Western blot analysis as described in the Fig. 1c. The signal intensities were quantified as described in the Materials and Methods. The signal intensities of pepsinogen C (pH 7.6), β-actin (pH 7.6) and full length AMCase (pH 7.6 and 2.0) at incubated 0 min were defined as 100%. The signal intensity of pepsin (pH 2.0) at incubated 5 min. CatD of AMCase were calculated as 100% at incubated 0 min of full length. as 100%. Values represent mean ± SD conducted in triplicate. $^*p < 0.05$, $^{**}p < 0.01$. P-values were determined using Student’s t-test.

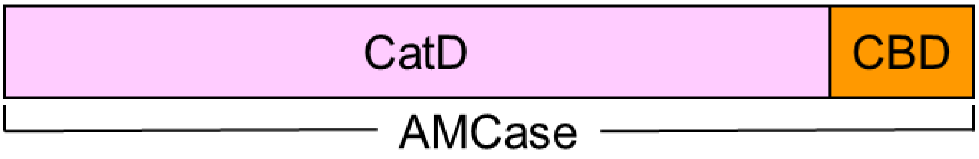

Supplementary Fig. S2. Schematic representation of mouse AMCase protein. Mouse AMCase is composed of an N-terminal catalytic domain (CatD) and a C-terminal chitin binding domain (CBD).

YNLICYFTNWAQYRPGLGSFKPDDINPCLCTHLIYAFAGMQNNEITTIEWNDV
TLYK**AFNDLKNRNSKL**KTLLAIGGWNFGTAPFTTMVSTSQNRQTFITSVIKFL
RQYGFDGLDLDWEYPGSRGSPPQDKHLFTVLVKEMREAFEQEAIESNRPRLMV
TAAVAGGISNIQAGYEIPELSKYLDFIHVMTYDLHGSWEGYTGENSPLYKYPT
ETGSNAYLNVDYVMNYWKNNGAPAEKLIVGFPEYGHTFILRNPSDNGIGAPTS
GDGPAGPYTRQAGFWAYYEICTFLRSGATEVWDASQEVPYAYKANEWLGYDNI
KSFSVKAQWLKQNNFGGAMIWAIDLDDFTGSFCDQGKFPLTSTLNKALGISTE
GCTAPDVPSEPVTTPPGSGSGGGSSGGSSGGSGFCAD**KADGLYPVADDRNAFW**
QCINGITYQQHCQAGLVFDTSCNCCNWP

Supplementary Fig. S3. Locations and sequences of the antigens used for the anti N-terminal or anti C-terminal AMCase antibody in the mature mouse AMCase protein. The antigen sequences are shown in red, and the CBD region is underlined. Deduced molecular masses of the CBD is about 5 kDa.

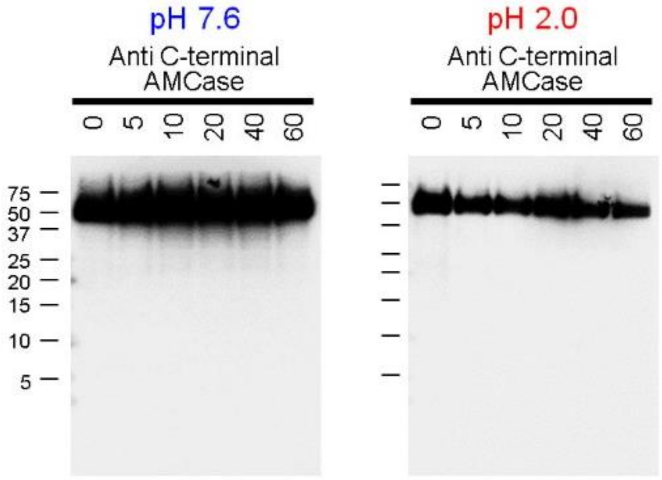

Supplementary Fig. S4. Western blot analysis of the AMCase using anti C-terminal antibody. Soluble proteins obtained from mouse stomach were incubated at 37ºC for 0, 5, 10, 20, 40 and 60 min at pH 7.6 or 2.0. The fragments of CBD (about 5 kDa) itself and the 9 kDa containing CBD were not detected at even longer exposure condition.

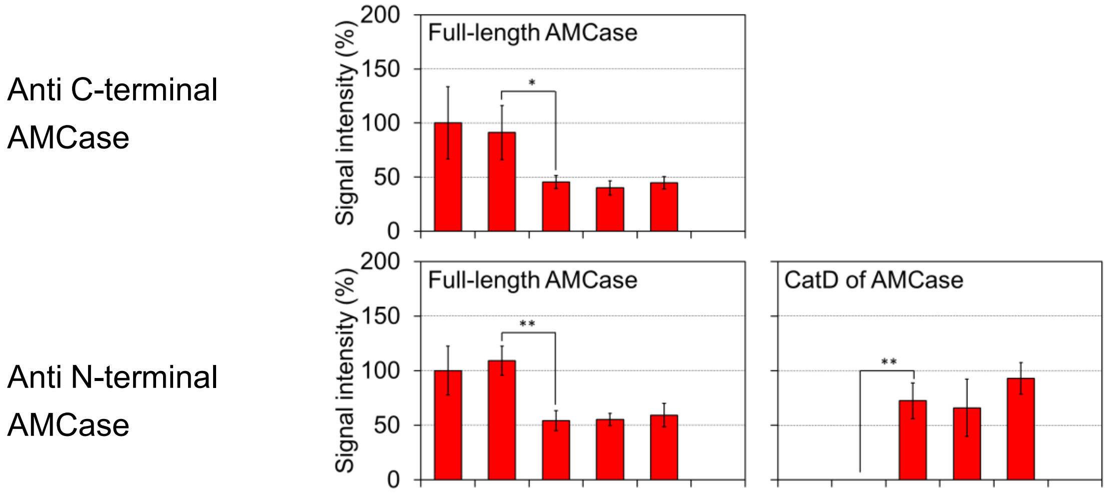

The extract of mouse stomach	+	+	+	+	+	-	+	+	+	+	+	-
Trypsin/Chymotrypsin	-	-	-	1	50	+	-	-	-	1	50	+
Incubation time at pH 2.0 (hr)	-	0	1	1	1	1	-	0	1	1	1	1
Incubation time at pH 7.6 (hr)	-	-	-	3	3	3	-	-	-	3	3	3

Supplementary Fig. S5. Changes of AMCase protein levels in the Western blot in Fig. 3. The signal intensities in Fig. 3b and c were quantified as described in the Materials and Methods. The signal intensities of full length AMCase at incubated 0 min were defined as 100%. Values represent mean ± SD conducted in triplicate. *$p < 0.05$*, *$^{**}p < 0.01$.* P-values were determined using Student's t-test.

GTGGATTCTGTGCCGACAAAGCAGATGGCCTCTACCCTGTGGCAGATGACAGAAATGCT
TTTTGGCAGTGCATCAATGGAATCACATACCAGCAGCATTGTCAAGCAGGGCTTGTTT**T**
TGATACCAGCTGTAATTGCTGCAACTGGCCA***AGATCT***TGGCAACCAGGCCTCTGGCTGG
TGCTCCTCCTCTGGC**TGCCAAGGCATTGTAGACACA**GGCACCTCTCTGCTCGTCATGCC
TGCCCAGTACCTG**AATGAACTTCTGCAGACCATAGGAG**CCCAGGAAGGAGAGTATGGAC
AGTATTTTGT***CTCGAG***TGGACTTGGATGACTTCAAGGGTTCCTTCTGCAACCAGGGCCC
GTACCCTCTCATCCGGACACTA**CGGCAGGAACTAAATCTTCCAT**CCGAGACTCCAAGGA
GCCCAGAACAGATA**ATACCTGAGCCACGCCCA**TCTTCTATGCCAGAGCAGGGACCCAGC
CCAGGGCTA***CTGCAG***GAGCTGAACGGGAAGCTCACTGGCATGGCCTTCCGTGTTCCTAC
CCCCAA**TGTGTCCGTCGTGGATCTGA**CGTGCCGCCTGGAGAAACCTGCCAAGTATGATG
ACA**TCAAGAAGGTGGTGAAGCAGG**CATCTGAGGGCCCACTGAAGGGCATCTTGGGCTAC
ACT***GCGGCCGC***CGAGCAGGAGATGGCCACTGCCGCATCCTCTTCCTCCCTGGAGAAGAG
CTATGAGCTGCCTG**ACGGCCAGGTCATCACTATTG**GCAACGAGCGGTTCCGATGCCCTG
AGGC**TCTTTTCCAGCCTTCCTTCTTG**GGTATGGAATCCTGTGGCATCCATGAAACTACA
TTCAATTCCATCATGAAGTGTGACGTTGACATCCGTAAAGACCTCTATGCCAACACAGT
GCTGTCTGGTGGTACCACCATGTACCCA***GAATTC***CTCCT**GCAGAGACCCTGGAAGTAAA**
GTGTCCACTCTACTGAAGAAAATGGAGAACTGGTCACCTTCAAGCCCGGGTG**CTGAATC**
CTCGGCCTTCTATGGGCCTCTATG**CGATGTGTAGCCAGGATTGT**CAGGTGGTCAAGAGG
GTTAGTCAAGACTGCCCCTCCACCATGCCACCCCCTGCGACAACT**CTATCTACATCTAC**
CACGCCACCTGTCGGCCC**CAGGGTCCTCAACACTTCATAG**CAGACCTGTCCAAGAAGCA
GAGGCCACGAATGGAGGAAGAAGAAGAGGC**CTACGGATGGATGGACTTTGG**CCGCCTTC
TG**CCAGCTCTACCTGAATGAGAAG**GACTACCCACCTGGCTATGCCTTTGATGTGGAGGC
CATGAACTTTCCGAGTAGTGGCCTCTG**CTTTGCGGACTTGTATCCA**TGAT

Supplementary Fig. S6. Nucleotide sequence of the single standard DNA used for real-time PCR. The single standard DNA, 1,349 nucleotides long, contained cDNA fragments of AMCase, pepsinogen C, Chit1, GAPDH, β-actin, gastric intrinsic factor, mucin, gastrin and H^+/K^+-ATPase in a one-to-one ratio. The PCR-target regions are shown in bold underline. The restriction sites are shown bold italic. Red, AMCase; green, pepsinogen C; blue, Chit1; purple, GAPDH; orange, β-actin; pastel pink, gastric intrinsic factor; rose berry, mucin; aqua, gastrin; light blue, H^+/K^+-ATPase. Calculated molecular mass, 833480.6.

Supplementary Table S1. List of qPCR primers. Gif, gastric intrinsic factor; ATPase, H^+/K^+-ATPase.

Target gene	Fw (5' to 3')	Rv (5' to 3')
Gif	GCAGAGACCCTGGAAGTAAAG	CATAGAAGGCCGAGGATTCAG
Mucin	CGATGTGTAGCCAGGATTGT	GTGGCGTGGTAGATGTAGATAG
Gastrin	CAGGGTCCTCAACACTTCATAG	CCAAAGTCCATCCATCCGTAG
ATPase	CCAGCTCTACCTGAATGAGAAG	TGGATACAAGTCCCGCAAAG
